# A novel frameshift mutation of Chediak-Higashi syndrome and treatment in the accelerated phase

**DOI:** 10.1590/1414-431X20165727

**Published:** 2017-03-23

**Authors:** X.L. Wu, X.Q. Zhao, B.X. Zhang, F. Xuan, H.M. Guo, F.T. Ma

**Affiliations:** Department of Pediatric Hematology-Oncology, The Second Hospital of Hebei Medical University, Shijiazhuang, China

**Keywords:** Chediak-Higashi syndrome, Accelerated phase, Gene sequencing, Therapy

## Abstract

Chediak-Higashi syndrome (CHS) is a rare autosomal recessive immunodeficiency disease characterized by frequent infections, hypopigmentation, progressive neurologic deterioration and hemophagocytic lymphohistiocytosis (HLH), known as the accelerated phase. There is little experience in the accelerated phase of CHS treatment worldwide. Here, we present a case of a 9-month-old boy with continuous high fever, hypopigmentation of the skin, enlarged lymph nodes, hepatosplenomegaly and lung infection. He was diagnosed with CHS by gene sequencing, and had entered the accelerated phase. After 8 weeks of therapy, the boy had remission and was prepared for allogenic stem cell transplantation.

## Introduction

Chediak-Higashi syndrome (CHS) is a rare autosomal recessive disorder characterized by variable degrees of oculocutaneous albinism, recurrent infections, a tendency for mild bleeding, and progressive neurologic deterioration (1). The first case of the syndrome was reported in 1943, and <500 cases have been reported worldwide for the past 20 years (2). In China, no more than 50 cases have been reported over the past decades. The genetic defect that results in CHS was identified in 1996, and was mapped to human chromosome 1q42–44 (3). The CHS gene was originally called LYST, which contains 53 exons with an open reading frame of 11,406 bp, and encodes for a 3801 amino acid protein, CHS1. Although the exact function of CHS1 remains unknown, it has been thought to play a role in regulating lysosome-related organelle size, fission and secretion (4).

The ‘accelerated phase’ of CHS, namely, hemophagocytic lymphohistiocytosis (HLH), develops in 50-85% of cases, and is fatal if not treated because of multi-organ dysfunction. Hence, the treatment of this rare disorder is also complicated and difficult, especially in the accelerated phase.

Here, we report the case of a young boy with a novel frameshift mutation inherited from his parents, who presented to our clinic in the accelerated phase of CHS and received 8 weeks of therapy.

## Case Report

**Figure 1:**
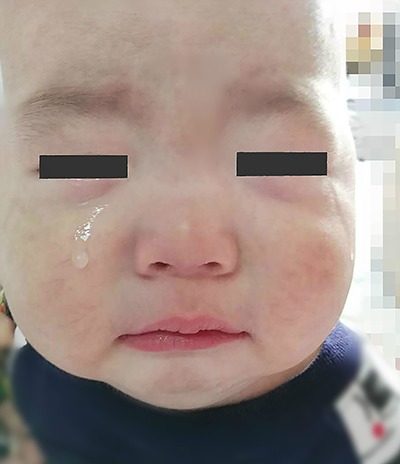
Oculocutaneous albinism and patchy hypopigmentation of the skin of a 9-month old boy with Chediak-Higashi syndrome.

A 9-month-old boy, who was born from a non-consanguineous marriage, presented to our clinic with complaints of continuous high grade fever for the past 15 days. His body temperature was over 39.5°C in spite of ibuprofen/acetaminophen and anti-infection therapy. The development of his body frame was normal, weighing 10 kg. Clinical examination revealed mild pallor, gray hair and patchy hypopigmentation of the skin (Figure 1), red rashes on the trunk, submandibular lymphadenopathy and protuberant abdomen with massive hepatosplenomegaly. The boy had no mucocutaneous bleeding and iris pigmentation, and cardiovascular and nervous systems were normal. His history since birth was negative for frequent chest infections, draining bilateral ears and fever. There was no family history of the disease.

### Investigations

**Figure 2:**
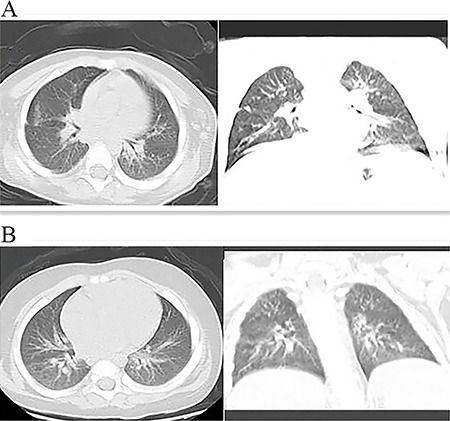
*A,* Double pneumonia with multiple patchy lesions before therapy. *B*, Decrease of pulmonary inflammation after therapy.

Routine blood examination revealed pancytopenia with febrile neutropenia (hemoglobin of 72 g/L, white blood cell count of 9.05×10^9^/L, neutrophil count of 0.9×10^9^/L, and platelet count of 43×10^9^/L). Blood biochemical examination revealed elevated C-reactive protein (69.4 mg/L), high ferritin levels (>2000 ng/mL), low fibrinogen levels (0.66 g/L), hyponatremia (129.5 mmol/L), hypoalbuminemia (20.1 g/L), and hypertriglyceridemia (4.99 mmol/L). Relevant hematological findings were LDH 1062 U/L, ALT 92.8 U/L, AST 339.1 U/L, GGT 350 U/L, and TBA 241.4 μmol/L. Routine urine and cerebrospinal fluid examination were normal. Blood cultures were negative. Epstein-Barr virus (EBV) DNA detection was 1.34×10^6^ IU. Abdominal ultrasound revealed hepatosplenomegaly. Lung CT images revealed double pneumonia with multiple patchy lesions (Figure 2).

**Figure 3:**
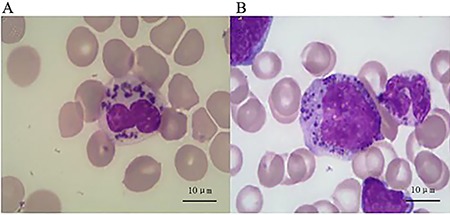
Abnormal intracytoplasmic giant granule in granular leukocytes in peripheral blood smear (*A*) and in their bone marrow precursors (*B*) by microscopic examination.

**Figure 4:**
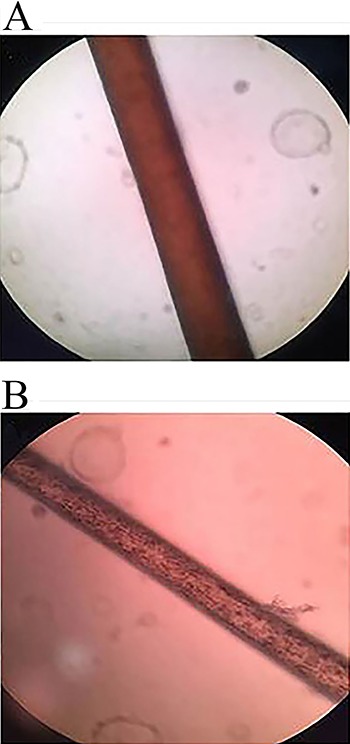
Comparison of normal hair (*A*) and Chediak-Higashi syndrome patient’s hair (*B*). Light microscopic images of the boy’s hair show abnormal clumping of melanin. Pigment clumps were small and uniformly distributed along the hair shaft.

Peripheral blood smear revealed several abnormal giant granules in most leukocytes. Bone marrow aspirate revealed prominent granules within the lymphocytes and myeloid cells (Figure 3). Furthermore, hemophagocytosis was observed on the bone marrow aspiration smear. Light microscopic images of the boy’s hair revealed abnormal clumping of melanin. Pigment clumps were small and uniformly distributed along the hair shaft (Figure 4).

**Figure 5:**
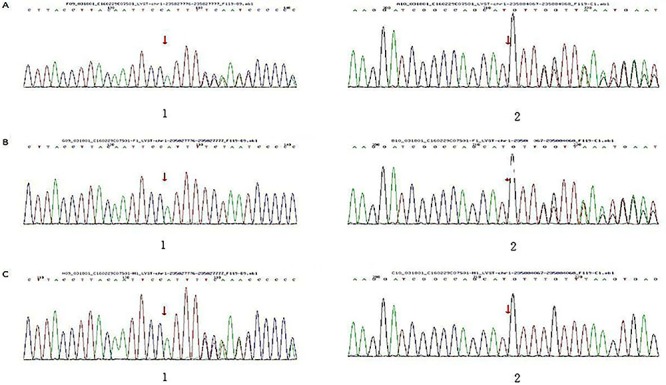
Gene mutations of a 9-month old boy with Chediak-Higashi syndrome and of his family. *A*, The boy's gene mutations at c.11183delA, p.N3728fs (1)/c.9453delA, p.K3151fs (2). *B*, The father's gene mutations at c.9453delA, p.K3151fs (2). *C*, The mother's gene mutations at c.11183delA, p.N3728fs (1).

HLH-related genes (including *AP3B1*, *FASLG*, *PRF1*, *TNFRSF1A*, *BIRC4*, *ITK*, *RAB27A*, *UNC13D*, *CASP10*, *ITPKC*, *SH2D1A*, *CD27*, *LYST*, *STX11*, *FAS*, *MAGT1* and *STXBP2*) were detected with high-throughput sequencing. It was found that *LYST* gene mutations at c.11183delA, p.N3728fs and c.9453delA, p.K3151fs were separate from his mother and father. Based on HGMD (Human Gene Mutation Database) analysis, these were novel frameshift mutations, validated by Sanger sequencing (Figure 5 and Table 1).

**Table 1:**
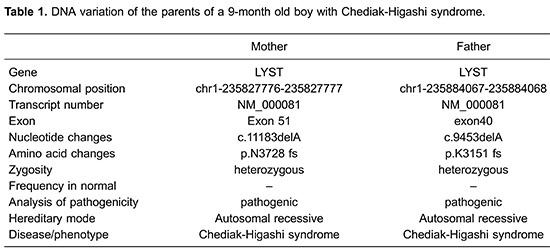
DNA variation of the parents of a 9-month old boy with Chediak-Higashi syndrome.

### Treatment

The boy was diagnosed with CHS in the accelerated phase due to his typical clinical characteristics, laboratory examinations and the detected LYST gene. According to the 2004 revised guidelines of the Histiocyte Society (5), the patient fulfilled the diagnostic criteria for HLH (five out of eight criteria). The HLH-2004 treatment protocol, including cyclosporine A (5 mg·kg^-1^·day^-1^), dexamethasone (10 mg·(m^2^)^-1^·day^-1^, half dose every 2 weeks), and etoposide (150 mg/m^2^, twice a week for the first 2 weeks and once a week during the following 6 weeks), was administrated (5). Maximal supportive care was provided, such as antibacterial and antiviral treatment (meropenem and ganciclovir), liver and cardiac protective agent, high doses of intravenous gamma globulin (2 g/kg), multiple transfusions, including platelets and packed red blood cells for anemia and fresh frozen plasma to improve the function of blood coagulation, prophylactic oral cotrimoxazole, and antimycotic voriconazole (6). After the initial 8 weeks of therapy, all blood parameters returned to normal, pneumonia was cured (Figure 2), and the disease was in temporary remission. Continuation therapy of the HLH regimen was then started. To date, the boy is in good condition. Stem cell transplantation has been planned.

## Discussion

Chediak-Higashi syndrome is a rare lysosomal storage disorder caused by mutations in the lysosomal trafficking regulator (LYST), which encodes the CHS1/LYST protein (7,8 [Bibr B09] [Bibr B10] [Bibr B11]). CHS1/LYST is a 430-kDa protein with several distinct domains implicated in various aspects of vesicular trafficking, but its exact function remains to be elucidated. It has been thought to play a role in regulating lysosome-related organelle size, fission, and secretion (4). To date, 63 CHS1/LYST mutations have been described, including 31 substitutions (20 nonsense, 11 missense), 19 deletions, 9 insertions, and 4 acceptor splice sites (7–13). Early reports that indicated frameshift show that nonsense and splice site mutations resulting in an absent CHS1/LYST protein correlate with severe childhood CHS, while milder adolescent or adult forms of CHS present with at least one missense mutation, probably encoding a partially functioning protein. (12,14). In our case, we found novel compound heterozygous frameshift mutations from his parents. These mutations may have been responsible for the infancy onset with severe manifestations.

Typical clinical presentations of CHS usually exhibit varying degrees of skin, eyes and hair hypopigmentation, frequent infections, progressive neurologic deterioration, and a tendency for mild bleeding (1). Hair color may be blond, gray, or white, and is often distinguished with a silvery or metallic sheen. Microscopic examination of the hair can also reveal clumped melanin granules, which are small and uniformly distributed along the hair shaft (15). Iris hypopigmentation may be associated with decreased retinal pigmentation, and ocular manifestations include photophobia, decreased visual acuity, nystagmus and strabismus. Oculocutaneous albinism and patchy hypopigmentation of the skin or speckled hyperpigmentation may commonly be seen in some patients. Increased susceptibility to infection, especially in the skin and respiratory tract, is due to the defective function of neutrophils, poor mobilization from the bone marrow, decreased deformability resulting in defective chemotaxis, and delayed phagolysosomal fusion resulting in impaired bactericidal activity (16). Staphylococcus and Streptococcus are the most frequently isolated species from these infection sites (16). Patients also show a mild bleeding tendency that often manifests as bruising and mucosal bleeding as a result of defective platelets. However, this does not usually require treatment.

CHS is classified into three phenotypes based on clinical symptoms: childhood, adolescent and adult forms (12). The childhood form accounts approximately for 80–85%. It is characterized by frequent severe infections and massive HLH, also termed as the accelerated phase. The basic pathophysiological mechanism underlying HLH is inappropriate cytotoxic activity, which leads to the impaired downregulation of immune responses and sustained activation and proliferation of cytotoxic T lymphocytes and NK cells (15,17). It often occurs following initial exposure to EBV, and has a high mortality rate. The diagnosis of HLH must be based on clinical and laboratory criteria, according to the 2004 guidelines of the Histiocyte Society, which include continuous fever, lymphadenopathy and hepatosplenomegaly, cytopenias that affect at least two or three cell lineages, hypertriglyceridemia and/or hypofibrinogenemia, hemophagocytosis, low NK-cell activity, hyperferritinemia, and high soluble interleukin-2-receptor (soluble CD25) levels (5). It is worth stressing that the presence of five of the eight criteria confirm HLH. Additionally, there are secondary characteristics, such as skin rash, jaundice, edema, pleural or pericardial effusions, hypoproteinemia and hyponatremia, and elevated transaminases and lactate dehydrogenase (18). Genetic defection is necessary for the diagnosis and treatment of HLH. At present, our laboratory was able to detect 17 HLH-related genes, including the latest gene reported in literature. Through this approach, some severe gene mutations were found. Hence, hematopoietic stem cell transplantation (HSCT) should be planned early. However, approximately 15–20% of patients follow a less severe clinical course of CHS, which are the adolescent and adult forms. Children with these forms present with a lower frequency of infection during childhood, and survive until adulthood without experiencing the accelerated phase. Nonetheless, during adolescence or adulthood, they develop progressive neurologic symptoms including intellectual deficit, dementia, peripheral neuropathy, parkinsonism, balance abnormalities, and tremors.

In addition to the above presentations of CHS, we frequently observe giant granules in the peripheral leukocyte and in their bone marrow precursors by microscopic examination. This typical and exclusive phenomenon must be distinguished from the other two autosomal recessive transmission syndromes, the Griscelli syndrome and Elejalde syndrome. Both are characterized by skin hypopigmentation, silvery gray hair, central nervous system dysfunction in infancy and childhood, and very large and unevenly distributed granules of melanin in the hair shaft and skin.

The main treatment for CHS focuses on three fields: supportive management of disease-derived complications, treatment of the accelerated phase or HLH, and HSCT, which has been recognized as the most effective treatment for hematologic and immune defects caused by CHS. However, it cannot improve progressive neurologic dysfunction. Tardieu reported three patients who survived for 20 years after unrelated hematopoietic stem cell transplantation (19). Without exception, these cases gradually presented with severe central nervous system symptoms including balance abnormalities, tremors, intellectual deficit, dementia, peripheral neuropathy and cerebellum atrophy. A retrospective review of 35 cases of HSCT in CHS patients indicated a 5-year overall survival of 62%.

The HLH-2004 treatment protocol recommends an 8-week induction therapy with corticosteroids, etoposide, and cyclosporine A. Intrathecal therapy with methotrexate and prednisone is restricted to patients with evidence of central nervous system disease progression after 2 weeks of systemic treatment, or in patients with worsening or unimproved cerebrospinal fluid pleocytosis (5). Early diagnosis and early treatment is very important to reduce the mortality rate of HLH.

Analysis of patients who underwent the previous HLH-94 protocol indicated that prognosis without treatment was poor, with a median survival of 1–2 months. A meta-analysis (20) conducted in China revealed that among the 29 patients with CHS and HLH, 9 received chemotherapy with a 100% improvement rate. However, among the other 20 patients that only received ordinary antibiotic therapy, 11 died. The onset of our patient was at 9 months of age, and presented with the prompt progress of HLH symptoms. After symmetric and comprehensive examination within 48 h, he received a high-dose dexamethasone and etoposide treatment. Oral cyclosporine A was administrated 2 weeks later due to poor compliance. His temperature decreased within 72 h and normalized within 7 days. All blood examination results returned to normal approximately 3–4 weeks later. We are actively engaged in looking for a matched donor during the therapeutic period. The age of onset, the progress of the illness, and the presence of HLH are the critical factors for determining whether chemotherapy should be performed.

In recent years, the absence or decreased intensity of CD107a measured on the cell surface by flow cytometry have high sensitivity and specificity for the diagnosis of the granule exocytosis primary disorder, which has been verified in CHS patients. Patients with absent CTL cytotoxicity have an indication for early HSCT due to the high risk of developing HLH. Further investigation is expected to confirm such findings, and put forward approaches to significantly improve the treatment effect.
